# An assessment of early diagnosis and treatment of malaria by village health volunteers in the Lao PDR

**DOI:** 10.1186/1475-2875-9-347

**Published:** 2010-12-01

**Authors:** Viengvaly Phommanivong, Khanti Thongkham, Gopinath Deyer, Jean P Rene, Hubert Barennes

**Affiliations:** 1 Institut de la Francophonie pour la Médecine Tropicale, Vientiane, BP 9519, Lao PDR; 2 World Health Organization, Vientiane Lao PDR

## Abstract

**Background:**

Early diagnosis and treatment (EDAT) is crucial to reducing the burden of malaria in low-income countries. In the Lao PDR, this strategy was introduced in 2004-2005 and an assessment was performed at the community level in January 2007.

**Methods:**

EDAT with malaria rapid diagnostic test (MRDT) and artemisinin combination therapy (ACT) was prospectively assessed among 36 randomized village health volunteers (VHVs) and 720 patients in six malaria-endemic provinces of Laos (three pilot provinces (PP), and three non-pilots provinces (NPP)). ACT was also retrospectively assessed among 2188 patients within the same areas from June to November 2006. Two checklists were used and scores were calculated.

**Results:**

EDAT performance of the VHVs was rated better in PP than in NPP (16.67% versus 38.89%, respectively, p = 0.004). Nearly all VHVs could diagnose malaria but only 16 (44%) could describe the symptoms of severe malaria. In January 2007, 31/720 (4%) patients tested positive using the Paracheck^® ^test, 35 (5%) with microscopy (sensibility: 74.3%, specificity 99.3%, positive and negative predictive values: 83.9% and 98.7%, respectively). Patients from June to November were at higher risk of malaria: 35.19% of 2,188 febrile patients were positive (OR: 10.6, 95%CI: 7.4-15.5, p < 0.000). VHVs reported the MRDT easy to use, and yielded a satisfactory performance score. EDAT performance was rated as poor despite satisfactory results regarding ACT treatment, duration and dosages. Pre-referral treatment of severe malaria was infrequent and often inadequate, with 20% of these patients dying. Results suggest a higher mortality from severe malaria than officially reported. Shortage of ACT was frequent.

**Discussion and conclusion:**

MRDT and ACT are useful and efficient and can be used by VHVs. VHVs' global EDAT performance is enhanced through training and monitoring. Persistent gaps in knowledge, care of patients and wrong treatment have to be addressed.

## Background

Half of the world's population is at risk of malaria, and among the 243 million clinical cases every year, the majority occurs in the world's poorest countries [1]. This is not an inevitable burden. In the past decade, there have been considerable advances in the cost-effectiveness of preventing and treating malaria, together with an increasing commitment from donors and affected countries to combat this disease. However, life-saving measures continue to be beyond the reach of the majority of people who need them. Among strategies, early diagnosis and treatment (EDAT) has been recommended [2].

In the Lao People's Democratic Republic (Lao PDR), with a current estimated population of 6 million, malaria is considered endemic throughout the country. However, intensity of transmission varies between different ecological areas ranging from 0.001 to 32.7 per thousand infected bites and according to the seasons [3]. Around 60% of the population is estimated at risk [3].

After reports of increasing resistance to chloroquine and sulphadoxine-pyrimethamine, the national malaria treatment guidelines were changed to artemisinin-based combination therapy (ACT) (artemether/lumefantrine, Coartem^®^) in 2004 [4-6] and malaria rapid diagnostic tests (RDT) were introduced. This early diagnosis and treatment (EDAT) strategy was initially implemented in early 2005 at the village and health centre level in three pilot provinces (PP) (Sekong, Saravan and Attapeu) covering a total of six districts and 60 villages. Two village health volunteers (VHVs) per village were selected and trained. Following the pilot phase, EDAT was adopted as a national strategy and extended to all 17 provinces.

An evaluation of a short training on MRDT procedure of 64 VHVs had shown a good reliability in 2003 [7]. However, previous studies indicated that RDT accuracy was highly user-dependent despite its apparent simplicity. Basic diagnostic tests risked being performed and interpreted poorly, but by providing simple and clear instructions these errors could be reduced, thus increasing their accuracy from 70% to 80% [8].

This study was conducted more than one year after the initial expansion of the EDAT strategy in six endemic provinces of Lao PDR. Its objective was to evaluate the effects of training initiated in the three pilot areas in 2005 compared to the training in the expanded phase for non-pilot provinces: performing an RDT test, its interpretation and prescribing behaviour of the malaria volunteers.

## Methods

### Malaria in the Lao PDR

In 2009, only five deaths among 22 784 confirmed cases of malaria were reported compared with 600 deaths and 70 000 confirmed cases in 1997 [9]. The mortality rate declined over the past 10 years from 0.12 per thousand in 1996 to 0.01 in 2006. *Anopheles dirus*, *Anopheles minimus *and *Anopheles maculatus *are the main vectors, and *Anopheles jeyporiensis *and *Anopheles nivipes *the secondary ones [10]. The National Malaria Control Programme (NMCP) is based on Roll Back Malaria strategies. The main preventive strategy is the use of insecticide-treated nets (ITN) [9]. By 2007, operational coverage of ITN was estimated to reach half of the population at risk. RDT and ACT are provided free of charge through the public health facilities [9].

### Malaria village health volunteers

The village health volunteers (VHVs) constitute the most peripheral level of the public health care system in Lao PDR. Volunteers provide primary health care services, including diagnosis and management of basic diseases (respiratory diseases, diarrhoea, and uncomplicated malaria) as well as performing health education, assist in vaccination campaigns and insecticide treatment of bed-nets, and reporting of morbidity and mortality data to health or district health offices [3]. VHVs (not malaria-specific volunteers but primary health care workers) are selected by the village committee in consultation with villagers, the Community Health and the District Health Office. Selection is based on pre-determined criteria. Often the criteria cannot be met and the best person for the role is selected. Each village is required to have two volunteers. But there is often only one person who has the literacy skill to be a VHV in rural areas.

VHVs play a pivotal role in malaria control:

• Village data collection

• Monthly reports to health centre

• Health education and community mobilization campaigns

• Diagnosis and treatment

• Distribution and re-dipping of ITN [11].

### Training of VHVs on EDAT

The EDAT strategy was first introduced in 20 villages/districts of three districts of the three pilot provinces in 2005. The training covered both health staff at district hospitals, health centres within the catchment areas of the target villages and VHVs (2 per village). The training was done mainly with trainers and facilitators from the central level. In subsequent years, between 2006 and 2008, the national programme gradually scaled up the coverage of RDTs and ACTs to more provinces, districts, health centres and villages. The cascade training involved trainers and facilitators from provincial level who attended training of trainers' sessions organized at the central level. The VHVs training took place over three days, which also included training in ITN distribution and use. In some provinces, pre- and post-tests were done although results of these were not available to the authors.

### Cross sectional study

**Figure 1:**
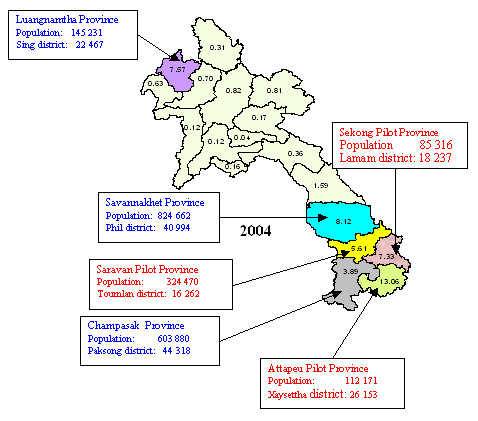
**Study sites and annual parasite incidence in Laos**. Adapted from 2004 CMPE data. Red is pilot provinces, blue is non pilot provinces.

Due to budgetary issues and time constraints, a cross sectional study was performed in six provinces from January to February 2007. The first three pilot provinces (PP) (Sekong, Saravan, Attapeu) were selected. Of the 14 other provinces in the country, three other non-pilot provinces (NPP) (Luangnamtha, Savanakhet and Champassak) were selected because nationally they had the highest retrospective incidence of malaria (Figure 1). Then a two-stage random sample procedure was applied. One district per province and six villages in each district were selected to make up a total of 36 villages.

The total population of the six provinces was 2.1 million. Of these, 10 200 (4.8%) and 11 700 (5.5%) people respectively, were suspected of, or confirmed with malaria in 2005 (2005 NMCP, unpublished data). At the village level, VHVs were included in this survey if they had been trained on ACT/RDT with the malaria programme, had more than six months experience and, if ACT/RDT was theoretically available in the village, according to the provincial distribution list. In each village one out of the two VHVs was selected using a random table. A pretested questionnaire was used to assess the characteristics of VHVs, their knowledge of malaria, and to provide basic information on their activities related to patients and malaria.

The quality of practice of VHVs was assessed by observation performed by the investigators. Two checklists were used and scores were calculated as previously described [2,7]. Briefly:

- A standardized 18-item form with four sections (history, patient care, observation of diagnosis and treatment, information and advice) to evaluate the VHVs practice (Table 1). Each item was given one point. The score of VHVs practice was defined as: excellent >80% of total score, good: 60-80%, poor <60%.

**Table 1:** Score of VHVs care of RDT positive patients in Laos

	Non pilot area n = 7	Pilot area n = 24	*p*	Total n = 31	Score %
**Disease history score**	13	73	0.43	86	29

1. How many people have a fever in your family?	1	3		4	13
2. Where did you go when you were febrile?	1	1		2	6
3. Duration of fever (days)	2	9		11	35
4. Did you have other symptoms?	2	6		8	26
5. What did you do/Which medicine did you take?	1	6		7	23
6. Explain the treatment (dosage)	4	24		28	90
7. Period of treatment (duration/length)	2	24		26	84

**Patient's care score**	16	62	0	78	26

8. Inform on the disease	2	9		11	35
9. Inform on malaria prevention	2	7		9	29
10. Explain the diagnoses	6	22		28	90
11. Correct timing of ACT	6	24		30	97

**Observation score**	23	94	0.12	117	39

12. Explain how to take the drugs	6	23		29	94
13. Explain duration of treatment	5	23		28	90
14. Explain proper timing for children treatment	4	21		25	81
15. Give ART suppository to children under 10 kg	2	3		5	16
16. Give correct dose/day	6	24		30	97

**Counselling score**	1	15	0.07	16	5

17. Give the information about worsening symptoms	0	14		14	45
18. Explain side effects of drugs	1	1		2	6
**Total score**	53	244	0.03	297	
**Global score (%)**	44.54	59.80			56.36

Non pilot zones: Luang Namtha, Savannakhet, Champasak.

Pilot zones: Sekong, Saravan, Attapeu

- A checklist of 15 criteria to evaluate the RDT performance (Table 1). The total of good responses scored was 15. Using the checklist, observers noted whether the VHVs performed the steps correctly (= 1), incorrectly or did not perform it (= 0). The score for RDT was defined as bad = <5; insufficient = 5 - 10; good = 10 - 13; excellent = >13.

All villagers with symptoms potentially related to malaria were enrolled, with a minimum of 20 cases per VHVs. Patients were included on entrance to the VHVs home, if they had a history of fever of 2-3 days duration, and no history of anti-malarial therapy during the previous week.

### Laboratory testing

A finger-prick blood sample was collected from each case to prepare thick and thin smears, and for RDT. A maximum of 20 tests was recorded per VHV. RDT tests were performed at the volunteer's house. All RDT results were confirmed by one expert reader of the investigation team on site. The Paracheck^® ^Pf Rapid One Step device for *Plasmodium falciparum *histidine rich protein (Orchid Biomedical Laboratories, Goa, India) was used according to a previously described procedure [8].

Thick and thin blood films were made immediately after blood collection and stained with 10% Giemsa for ten minutes. Slides were further read by two expert microscopists at the Centre of Malariology, Parasitology and Entomology (CMPE) in Vientiane. All microscopists were unaware of the results of the RDT tests. A minimum of 200 consecutive fields was counted in the thick blood film before classifying a slide as negative. Parasites in thick blood films were counted against 200-500 white blood cells. The parasite density was estimated for 8,000 white blood cells. If malaria parasites were seen, species were identified, based on their morphological features on the associated thin smear. The microscopic reading at the CMPE was considered the gold standard. RDTs were, as possible, performed according to the VHVs normal practice. In fact, some more patients came when they heard of a medical team doing tests.

### Retrospective study

Anti-malarial prescriptions were retrospectively assessed by checking the VHVs' register books for the period June - November 2006.

### Data management and analysis

Data was entered in Epidata 3.1 freeware (Odense, Denmark) and cross-checked against original data sheets. Analysis was carried out with Stata, Version 8 (Stata Corporation, College Station, TX, USA). Fisher's exact test was used to assess associations between categorical variables, and Student's *t-*test for two normally distributed continuous variables. P < 0.05 was considered as significant.

Verbally-informed consent was obtained from all participants. The survey was performed with the permission of Lao national, regional and village authorities and in agreement with the Declaration of Helsinki [12]. Ethical approval from the National Ethical Review Board of Laos was not sought since the survey did not directly involve patient interventions beyond national MoH standard guidelines (Standard treatment guidelines) and routine national malaria programme activities (i.e. active case detection surveys are frequently carried out by the NMCP). Investigators were medical doctors from the Institut Francophone de Médecine Tropicale (IFMT) attending a master's course with special lectures on epidemiology, field research and public health. Pre-tests were performed before conducting the survey.

## Results

### VHV assessment

This study was performed in January 2007, during the dry season. Most of the 36 villages had very basic services: seven villages (19%) had a private pharmacy and 27 (75%) had a village drug kit provided by the Ministry of Health (which included only chloroquine, quinine, and traditional herbal medicines for malaria). Since the last distribution of ACT from the CMPE (on average seven months earlier) nearly half of the VHVs had a shortage of ACT (Table 2).

**Table 2:** Characteristics and knowledge of VHVs in six provinces of Laos, 2007

	Non pilot area n = 18 (%)	Pilot area n = 18 (%)	*p*	Total n = 36
Sex (female)	0 (0)	3 (16.67)	0.07	3 (8.33)
Age (years)*	34.66 (28.71-40.61)	38.22 (33.75-42.68)	0.3	36.44 (32.86-40.02)
Training on RDT* (days)	2.61 (1.99-3.22)	2.72 (2.07-2.92)	0.7	2.55 (2.19-2.91)
Number of training	1.55 (1.20-1.90)	3.33 (2.15-4.51)	0.004	2.44 (1.78-3.10)
Main indications of MRDT**				
-Fever	16 (88.89)	18 (100)	0.1	34 (94.44)
-Headache	14 (77.78)	10 (55.56)	0.1	24 (66.67)
-Chills	13 (72.22)	7 (38.89)	0.04	20 (55.56)
No drug shop in village	12 (66.67)	17 (94.44)	0.03	29 (80.56)
Use village's kit**	15 (66.67)	15 (83.33)	0.2	27 (75)
Received ACT since (month)*	5.94 (4.30-7.58)	8.05 (7.09-9.01)	0.02	7 (6.02-7.97)
ACT shortage****	7 (38.89)	10 (55.56)	0.31	17 (47.22)
**Treatment for uncomplicated malaria**^μ^				
-Chloroquine	14 (77.78)	4 (22.22)	0.004	18 (50.0)
-Coartem^®^	3 (16.67)	7 (38.89)	0.13	10 (27.78)
-Paracetamol	0 (0)	6 (33.33)	0.07	6 (16.67)
-Quinine	1 (5.56)	1 (5.56)	1	2 (5.56)
Knows where to get ACT	0	0		0
Cannot describe severe malaria^μμ^	11 (61.11)	5(50)	0.5	16 (44%)
Ever diagnosed severe malaria	7 (38.89)	8 (44.44)	0.7	15 (41.67)
Treatment given for severe malaria (n = 15)	3 (42.85)	6 (75)		9 (60)
- Referral	2 (28.57)	6 (75)	0.07	8 (55.33)
- Antimalarial	3 (42.85)	2 (25)	0.4	5 (33.33)
-Artesunate	3 (42.86)	0	0.03	3 (20)
-Quinine ^μμμ^	0 (0)	2 (25.5)	0.3	1 (6.67)
-Dextrose infusion alone	1 (14.28)	3 (37.5)	0.3	4 (26.67)

Non pilot areas: Luang Namtha. Savannakhet. Champasak.

Pilot areas: Sekong. Saravan. Attapeu.

*Mean and 95% IC = Interval confidence

** reported by VHVs

*** 73% of the VHVs will give chloroquine.

**** occurring since the initial provision of ACT and the survey

^μ ^Similar treatment options for suspected malaria (21 (58%) chloroquine; 9 (25%) Coartem^®^. 2 (6%) quinine; two associate chloroquine with Coartem^® ^or quinine).

^μμμ ^one received quinine + doxycycline

A total of 36 VHVs and their 720 patients with fever were assessed. Their characteristics are shown in tables 2 and 3. Of 720 patients, only 31 (4.3%) had a positive RDT. Except for fever, headache, and chills no other symptoms were reported to perform a RDT.

**Table 3:** Characteristics of 720 febrile patients seen by VHVs in 6 Lao provinces in January February 2007

	Patients n = 720 (%)
Sex ratio (F/M)	1.01
Mean age (years)	21.41
Below < 15 years	151 (20.97)

**RDT positive**	31 (4.3)
-< 5 years	6 (19.35)
-5-15 years	17 (54.83)
-> 15 years	8 (25.8)

The global performance of VHVs was better in PP than in NPP (Table 1, table 4). Of the 36 VHVs, 75% of them reported using the village's drug kit for malaria but none of these contained ACT. None knew where ACT could be obtained from, or what its cost was. ACT was prescribed infrequently either for confirmed or suspected uncomplicated malaria. VHVs from PP gave chloroquine more frequently than others (p = 0.004) (Table 2).

**Table 4:** Malaria rapid test (Paracheck®) evaluation in six provinces of Laos, 2007

N°	Paracheck^® ^Pf (Procedures)	Non pilot provinces n = 320	(%)	Pilot Provinces n = 320	(%)	*p*	Total n=720	(%)
1	Preparation	152	(42.2)	298.0	(82.8)	<0.001	450	(62.5)
2	Look at the expiry date	20	(5.6)	122.0	(33.9	<0.001	142	(19.7)
3	Open the package properly	315	(87.5)	327.0	(90.8)	0.1	642	(89.2)
4	Check desiccant and record	117	(32.5)	291.0	(80.8)	<0.001	408	(56.7)
5	Clean middle or ring finger with the alcohol pad	329	(91.4)	341.0	(94.7)	0.1	670	(93.1)
6	Wipe off alcohol with gauze	194	(53.9)	233.0	(64.7)	0.04	427	(59.3)
7	Prick finger with sterile lancet (pick the side of finger)	355	(98.6)	339.0	(94.2)	0.002	694	(96.4)
8	Discard blood lancet	211	(58.6)	257.0	(71.4)	<0.001	468	(65.0)
9	Squeeze one blood drop out	211	(58.6)	328.0	(91.1)	<0.001	539	(74.9)
10	Collect blood with pipette (touch the surface of the blood drop)	224	(62.2)	330.0	(91.7)	<0.001	554	(76.9)
11	Apply blood in sample well	230	(63.9)	340.0	(94.4)	<0.001	570	(79.2)
12	Discard pipette	241	(66.9)	266.0	(73.9)	0.05	507	(70.4)
13	Put buffer - 6 drops in 'B' well	283	(78.6)	340.0	(94.4)	<0.001	623	86.5
14	Wait for 15 min and interpret correctly	272	(75.6)	291.0	(80.8)	0.01	563	78.2
15	Record results	123	(34.2)	235.0	(65.3)	<0.001	358	49.7
	**Total score/15**	9.1		12.2			10.7	

Only 16 (44%) VHVs could describe the symptoms of severe malaria. Coma and convulsions were more frequently related with severe malaria by VHVs of PP (p = 0.09, p = 0.01). Only 15 (42%) had previously seen patients with severe malaria, three (20%) died, nine (60%) were treated by the VHVs and eight were referred to the hospital after the first treatment. Of these eight, three received artesunate injection, one oral quinine, one quinine with doxycycline, and four dextrose infusion only. Artesunate injections are not included in the village's kit.

### Patients' assessment

Of the 720 patients, 31 (4%) tested positive using the Paracheck^® ^test, versus 35 (5%) positive with microscopy; 31 (89%) *Plasmodium *identified were *Plasmodium falciparum*, and four *Plasmodium vivax *(Table 5). The sensitivity and specificity of the rapid tests for detecting *Plasmodium falciparum*, using the microscopy as a gold standard, were 74.3% and 99.3%, respectively. The positive and negative predictive values were 83.9% and 98.7%, respectively.

**Table 5:** Distribution of 720 suspected malaria patients after microscopy and MRDT in Laos, 2007

	Microscopy Positive n = 35 (4.8%)	Microscopy Negative n = 685 (95.2%)	Total n = 720 (100%)
**RDT**			
- Positive	26 (74.2)	5 (0.7)	31 (4.3)
- Negative	9 (25.7)	680 (99.2)	689 (95.69)
**Parasites**			
*-P. falciparum*	26 (74.28)	5 (0.72)	31 (4.3)
*-P. vivax*	0	4 (0.58)	4 (0.55)
Parasites mean density/μL*	618 (48-42400)	0	-

* Geometric mean (and range); 11 (37.11%) were below 500 parasites/μl

### RDT assessment

**Figure 2:**
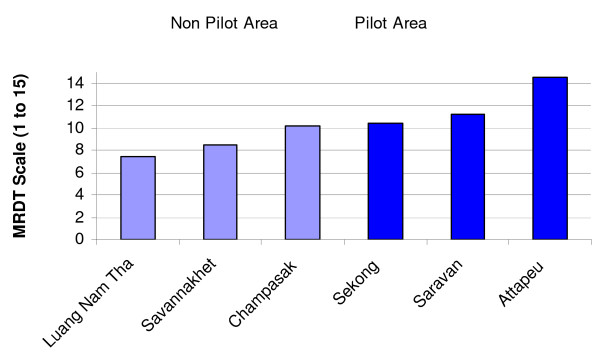
**Performance of VHVs using MRDT in 6 provinces of Laos**.

VHVs generally reported that RDT was easy to use. The mean global score rate of the different steps of RDT's procedure (10.7 on 15) was rated as good (Table 1). Two procedures related to checking expiry date and reporting of results were performed correctly by 50% of the respondents only. The VHVs from PP had an overall better score (12.2) than the others (9.1). Two NPP provinces (Luangnamtha and Savannakhet) yielded an insufficient score (Table 6 and Figure 2).

**Table 6:** Evaluation of malaria patients treated by VHV in Laos (register book June - November 2006)

	Non	Pilot	Provinces	(NPS)		Pilot	Provinces	(PS)	Total
	LNT n(%)	SVK n(%)	CPS n(%)	Total n(%)	SK n(%)	SRV n(%)	ATP n(%)	Total n(%)	n(%)
Patients tested	6	401	98	505	302	510	871	1683	2188
RDT positive (%)*	6 (100)	50 (12)	8 (8)	64 (12)	134 (44)	201 (39)	371 (43)	706 (42)	770 (35)
- < 5 years positive (%)**	0	12 (24)	0	12 (18)	29 (21.6)	7 (3.5)	304 (81.9)	340 (48)	352 (45.9)
ACT treatment (%)***	6 (100)	16 (33)	5 (62)	27 (42)	87 (65)	195 (97)	371 (100)	653 (92)	680 (88)

LNT: Luang Namtha, SVK: Savannakhet, CPS: Champasak, SK: Sekong, SRV: Saravan, ATP: Attapeu

RDT: rapid diagnostic test, ACT: arteminsin based combination treatment

* More patients tested positive in pilot provinces (p < 0.000)

** % of positive children under 5 years

*** Correct: Artesunate suppository for children and ACT for adult. More ACT treatment were given in pilot provinces (p < 0.000)

### ACT prescription for confirmed malaria

The overall mean performance was rated as poor (Table 1). VHVs of PP had a better performance than the others (score: 60% and 45%, respectively). Only one PP (Savannakhet Province) reached a satisfactory score (> 70%).

Of the 18 items, only eight were correctly answered (Table 1). The best performances were obtained regarding information on ACT dosage (97%) and treatment duration (97%). These items were related to the actual treatment given, which can be considered as fairly correct. Low scores were obtained with regard to disease history (29%) and patient information about worsening symptoms (45%) or side effects.

From June to November 2006, 2 188 febrile patients were tested, of which 35.19% were positive (Table 6). Patients testing positive for malaria ranged from 5.4% in NPP to 41.9% in PP, respectively (p < 0.001). They had a 10-fold chance of testing positive compared to January ((35.19% vs. 4.86%, OR: 10.6, 95%CI: 7.4-15.5, p < 0.001). Correct prescription of ACT ranged from 33% to 97% of confirmed cases. Two PP reached an excellent level of ACT prescription (Table 6).

## Discussion

The study evaluates the knowledge and practice of recently trained VHVs regarding the new EDAT malaria national strategy in the Lao PDR. It evaluates both the practice of RDT and the quality of ACT prescription of a sample of VHVs in 36 villages of six provinces. It confirms an overall satisfactory performance of RDT and supports the effectiveness and feasibility of RDT at the village level. It underlines persistent gaps in knowledge, care of patient and mistreatments among VHV as providers that have to be addressed. Overall, pilot provinces yielded better results than non-pilot provinces regarding care of patients, practice of RDT and ACT dispensation.

Using RDT, the survey also illustrates the decreasing rate of malaria morbidity at the village level in provinces considered as endemic. The incidence of confirmed malaria was low in northern, central and south-east provinces during the potential high malaria season. In the Lao PDR, the number of confirmed cases dropped from 53 156 in 1997 to 18 382, the year of the survey [1]. Transmission was probably very low and probably even stopped in January (dry season) in these regions [3]. By contrast, the incidence of malaria was persistent all over the year in the southern region. This justified the choice of these regions as pilot provinces. The remote locations of the survey at the village level probably reflect the reality of malaria since most of the patients had no other access to care. The population coverage was not calculated, which does not allow for estimating the incidence of malaria in these regions.

*Plasmodium vivax *represented 12.9% of confirmed malaria, a rate similar to that previously described, which suggests no relative decrease of this species in the Lao PDR, as experienced in Thailand [13,14]. However, the seemingly uniform lower prevalence of *vivax *malaria in endemic provinces might be partly because of the tendency of *P. vivax *to achieve and maintain lower-density parasitaemia, and the inverse relation between diagnostic sensitivity and parasitaemia count [15]. The detection of low level asymptomatic and mixed species infections with conventional light microscopy is limited and the RDT adopted in the Lao PDR does not diagnose *P. vivax *infection. Albeit the many challenges of introducing *P. vivax *diagnosis and treatment in Laos, the CMPE should consider a preliminary introduction of a *P. vivax *RDT.

VHVs are key persons to improving EDAT at village level in countries with poor health facilities. Until recently, most patients with fever were treated for malaria, based only on clinical suspicion by the VHVs, and most VHVs and pharmacy owners lacked basic knowledge of malaria management [2]. As a result, malaria tended to be overestimated and many patients were probably wrongly treated (infectious diseases such as rickettsia, typhoid or leptospirosis mimicking malaria are common in Laos) [16,17]. The survey supports the positive impact of training VHVs in PP, which yielded a generally better score of performance than others regarding both RDT assessment and ACT prescription. More training focussing on prevention of disease, worsening symptoms, and information of the patients are suggested for both groups of provinces.

Analysis within the provinces reveals a high discrepancy between them: only one province (Saravan) had an excellent score of achievement, but two out of the six had a very poor level. Comparing RDT at village level with the gold standard microscopy at the provincial level yielded predictive values above 80%. This confirmed the accuracy of RDT reading among the VHVs surveyed at the village level, but results were less optimistic than the previous survey, and warrants regular monitoring and quality control [7].

A possible limitation of this survey is that it was conducted during the low transmission season. The peak malaria transmission months in Laos are from July to October. However, the survey shows that malaria was still acute in two settings (Attapeu and Sekong) during the non-peak seasons. This study attempted to take this into consideration by including a retrospective survey covering six months of the transmission period. This later survey had the usual limitations of retrospective survey (incomplete files, omissions, inaccuracy).

To decrease the possibility of a bias due to the attractiveness of the medical team performing the survey, and the possibility of the VHVs showing their practice in their best light, VHVs were informed that they should continue their work as usual and that their identity would remain confidential. Nevertheless, the survey may probably have slightly overestimated the performance of VHVs, mostly for the first patients.

In fact, of the 720 febrile patients seen in January, only 4.8% had malaria parasites and 4.3% had a positive RDT. This potentially saves around 689 malarial treatments that would have previously been given in the absence of a confirmed diagnosis, and the use of ineffective chloroquine increasing the drug pressure [6]. The RDT strategy allowed to dramatically reduce the number of unnecessary cases of malaria treatment and potentially allows focusing better on other diseases. The benefits of RDT strategy were also well supported by the retrospective survey in the high malaria season, which revealed a 35% rate of malaria cases among the 2,188 suspected patients. This allowed the saving of 1 418 malaria treatments enabling the patient to save money, an important issue in a country were two-thirds of the population lives on less that USD 2 a day [18].

The low use of ACT despite the new policy is of concern. VHVs still prescribed non-ACT and ineffective chloroquine. Only a quarter of them would choose ACT. This signals the need for further information/training of VHVs to ensure compliance with the national treatment strategy.

Paracheck^® ^and Coartem^® ^are supplied not through the MoH village drug kits but through a separate vertical distribution system from the CMPE, which will probably improve after the first years of implementation. Timely reporting on consumption and stock levels by VHV may require an incentive program if out-of-stock situations are to be avoided.

Detecting symptoms of severe malaria and starting early treatment are key strategies to decreasing malaria related mortality. Despite recent training, VHVs still lacked knowledge on severe malaria detection and treatment [7]. Less than 20% of the VHVs identified 3-4 of the criteria of severe malaria. Only 41% ever diagnosed severe malaria. A total of 4 752 cases of malaria in 2006 were hospitalized (a proxy for severe malaria) in Laos, of which 3 910 (82%) were reported in the six surveyed provinces (CMPE, unpublished data). Since only half of the severe malaria cases were referred in the survey, this suggests reconsidering and probably doubling the estimation of severe malaria in Laos.

Additionally, in this study, the rate of mortality among severe malaria patients reached 20% at the village level. Hence, the figure of mortality obtained in only 36 villages suggests a probable underestimation of the official malaria deaths (21 reported deaths in 2006) [9].

Pre-referral treatment was only performed for half the severe malaria cases. Of them, only five (55%) received an anti-malarial treatment. Interestingly, injectable artesunate was not available in the village kits and the CMPE, as per guidelines, distributed this drug only to district and provincial hospitals. It can be hypothesized that some VHVs were given vials of artesunate from the CMPE programme. In fact, VHVs did not know where to get ACT or what it cost. They may also be vulnerable to ambulant drug vendors who sell fake or substandard artemisinin based anti-malarials, a major threat in Asia [19,20]. More studies should focus on the alternative treatments given at the village level and assess their effectiveness. VHVs need to know that counterfeit and sub-standard anti-malarials, including artesunate monotherapies, are widespread and that a safe source of ACT would be the national malaria programme. These results question the availability of potent anti-malarial treatment at the village level where one cannot expect a high rate of referral to a higher health care facility.

## Perspectives

RDT is easy to use, reliable and an appropriate test for use in the field by VHVs in the Lao PDR. Results also suggest more training to improve EDAT. Training and monitoring of user behaviour have to be reinforced especially in the provinces with poor success rates. Appropriate training and instruction have to be adapted to each province. Provinces with stable or decreasing transmission of malaria should be trained in the treatment of non-malaria fevers (dengue, arbovirosis but also infectious diseases such as leptospirosis, melioidosis, typhoid, rickettsia which are common but with scarce prevalence data at the national level) [16-18,21]. In endemic provinces the problem may be to scale up prevention and care of patients with appropriate diagnosis and early treatment. Current decrease of malaria and the emergence of dengue fever outbreaks have to be regularly assessed and monitored in the countryside.

## Conclusion

RDT and ACT, the new tools for EDAT in Laos are effective and well understood by VHVs. The survey confirms the ability of VHVs to use RDT, but underlines persistent gaps in knowledge, care of patients and treatment, which warrant further follow up, and training. The simplification brought by EDAT at village level will probably help the control of malaria, together with other strategy programmes.

## Conflict of interests

The authors declare that they have no competing interests.

## Authors' contributions

HB wrote the manuscript and is responsible for the overall coordination, design and analysis of the surveys in the Lao PDR. He is also guarantor. HB and GD designed the survey with the participation of PV and KT. PV and KT collected the data and performed the first analysis under HB's monitoring with regular advice from GD. HB performed the final analysis. GD, PV, KT, JPR and HB discussed the results. PV and KT wrote the first report with the participation of GD, JPR and HB. All authors contributed to the writing of the paper and read the final version.

## Funding

Global Fund against aids, tuberculosis and malaria through the CMPE of Lao PDR funded the survey
